# Pitfalls in Diagnosis: JMML versus *KMT2A* Rearranged Juvenile AML

**DOI:** 10.1155/2024/7151394

**Published:** 2024-09-06

**Authors:** Liesbeth Vanheeswijck, Sanjay Tewari, Robin Dowse, Nicola Potter, Jelena Jovanovic, Caroline L. Furness, Elsje Van Rijswijk

**Affiliations:** ^1^ Department of Paediatric Haemato-Oncology Royal Marsden Hospital NHS Foundation Trust, Sutton, Surrey, UK; ^2^ Department of Medical and Molecular Genetics King's College, London, UK

## Abstract

**Background:**

Lysine methyltransferase 2A (*KMT2A*) rearrangements are commonly found in juvenile acute myeloid leukaemia (AML). Although distinct diseases, there is a known clinical overlap between *KMT2A*-rearranged AML and juvenile myelomonocytic leukaemia (JMML). Both occur in infancy or early childhood and present with abnormal monocytosis. *Case Report*. We report a case of a 20-month-old girl, who presented with lethargy, recurrent infections, bruising, and marked hepatosplenomegaly. JMML was suspected after initial work-up, revealing an abnormal monocytosis without blast excess on immunophenotyping. The additional cytogenetic and molecular diagnostics, revealing a *KMT2A* rearrangement, was decisive for the confirmation of AML.

**Conclusion:**

This case highlights the challenges of diagnosing *KMT2A*-rearranged monocytic AML and the importance of careful morphological assessment in partnership with cytogenetic and molecular diagnostics to distinguish between *KMT2A*-rearranged AML and JMML. Moreover, the emerging role of molecular monitoring in AML is highlighted.

## 1. Introduction

Juvenile myelomonocytic leukaemia (JMML) is known as a myeloproliferative disorder of infancy and early childhood, with a median age of onset of 2 years [1]. It is a rare disease with an estimated incidence of 1.2 cases per million [1]. Typical presentation is with fever, splenomegaly, thrombocytopenia, and high white blood cell count with abnormal peripheral monocytosis [1]. Diagnosis relies initially on clinical and haematological features (Table 1) [3]; however, advances in genetic studies have now made a molecular diagnosis possible in nearly every patient with JMML [1, 4]. The 5^th^ edition of the World Health Organization (WHO) Classification of Haematolymphoid Tumours recently placed JMML in the group of myeloproliferative neoplasms and updated the diagnostic criteria (Table 1, Footnote) [2, 5].

Paediatric acute myeloid leukaemia (AML) is another rare disease, with an estimated incidence of 7 cases per million and a median age of onset of 6 years [6]. It is heterogenous in cytogenetic and genetic abnormalities, with lysine methyltransferase 2A (*KMT2A*) rearrangements accounting for 15–20% of paediatric AML [6]. These *KMT2A* rearrangements are predominantly associated with monocytic AML and occur more frequent in infants, with a median age of 2.2 years [6].

There seems to be some clinical overlap between *KMT2A*-rearranged AML and JMML, like the age of susceptibility and presentation with abnormal monocytosis [4, 7]. However, the pathogenesis and genetics are distinct, and both require different treatment strategies [7]. Hence, an accurate and timely diagnosis is vital.

## 2. Case report

**Figure 1:**
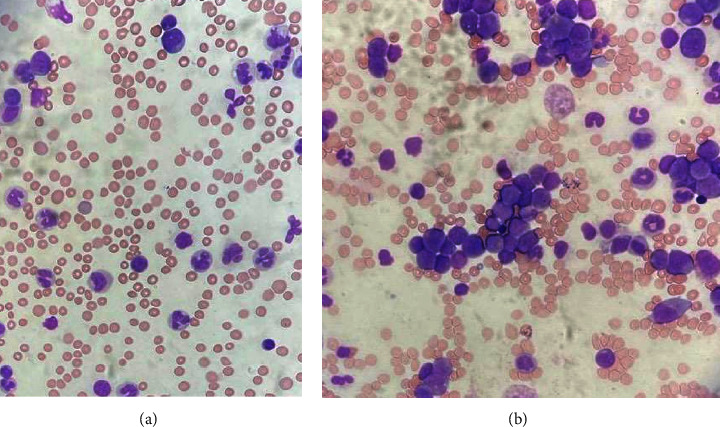
Photographs of peripheral blood and bone marrow smear. (a) Peripheral blood showed maturing granulocytic series with dysmorphic monocytes and all stages represented mature neutrophils. (b) Bone marrow aspirates showed a predominance of blasts and abnormal promonocytes morphologically with abnormal infiltrate identified as monocytic on immunophenotyping.

A previously healthy 20-month-old girl presented with a one-month history of being lethargic and having recurrent chest and ear infections, as well as a two-week history of bruising and petechiae on the skin. On clinical examination multiple bruising covering the arms, legs, and torso, as well as petechial spots widespread, were apparent. In addition, a marked hepatosplenomegaly was noted. The initial work-up showed a substantially increased white blood cell count of 203 × 10^9^/L, anaemia of 70 g/L, and a thrombocytopenia of 36 × 10^9^/L. Moreover, a high peripheral blood monocyte count of 16 × 10^9^/L was observed. Additional testing showed a slightly increased haemoglobin F level for her age (2.5%). Peripheral blood film (Figure 1) showed a leucocytosis with 40% of cells showing a combination of dysmorphic monocytes and promonocytes. There was no peripheral blast excess on immunophenotyping. Combined with the clinical presentation and given the patients' age, JMML was the likely diagnosis. FISH studies showed no evidence of *BCR*::*ABL1* and no evidence of monosomy 7 or deletion of 7q. Molecular studies showed likely pathogenic mutations in both *CBL* and *EZH2* genes. A likely pathogenic in-frame delins variant in *CBL*, c.1175_1185delinsCATGATAAGGATGTAAATGATAAGGATGTAAATGATAAGGATGTAACATGAGG p.(Lys392delinsThrTer) at 34% VAF, and a likely pathogenic frameshift variant in *EZH2*, c.2227_2231dup p.(Ile744MetfsTer25), VAF at 43.9% was detected by NGS. No other pathogenic/likely pathogenic variants were detected, more specifically no variants were detected in *KRAS*, *NRAS*, *PTPN11*, or *NF1* genes. The genome assembly GRCh37 was used for this analysis.

In the further diagnostic work-up, bone marrow aspirate (BMA) with trephine was performed, as well as a skin biopsy to investigate for *CBL* germline mutations. Morphological examination showed an atypical expansion of monocytes, but there were no immunophenotyping features to reliably differentiate malignant monocytic infiltrate from normal monocytes. The bone marrow trephine showed a hypercellular marrow with reduction in normal haematopoiesis and replacement by left shifted granulocytic population, frequent monocytes, and increased cells consistent with promonocytes. Immunohistochemistry showed an appreciable number of the cells to express the monocytic markers CD14, PGM1, weak CD33, and myelomonocytic marker KP1, and no cells were positive for the immature markers TdT, CD34, CD10, or CD117 and no excess of B or T lymphocytes. Initial trephine review was not diagnostically specific but considered compatible with potential clinical diagnosis of JMML by the reporting pathologist. However, morphological review of the bone marrow aspirate by an experienced haematologist reported a monocytic infiltrate with a predominance of blasts and abnormal promonocytes (Figure 1), highlighting a lack of consensus between reporting haematologists and pathologists. Subsequently, the *KMT2A* FISH test was urgently requested to complete diagnostic work-up and confirmed *KMT2A* gene rearrangement and hence classifying the case as *KMT2A*-rearranged AML.

Since there were no aberrant immunophenotyping markers to distinguish malignant from normal mature monocytes, immunophenotyping had no additional utility in this case. Consequently, as there was no leukaemia-associated immunophenotype for flow minimal residual disease (MRD) monitoring, a repeat BMA was performed, necessary for the validation of a patient-specific molecular marker work-up.

**Figure 2:**
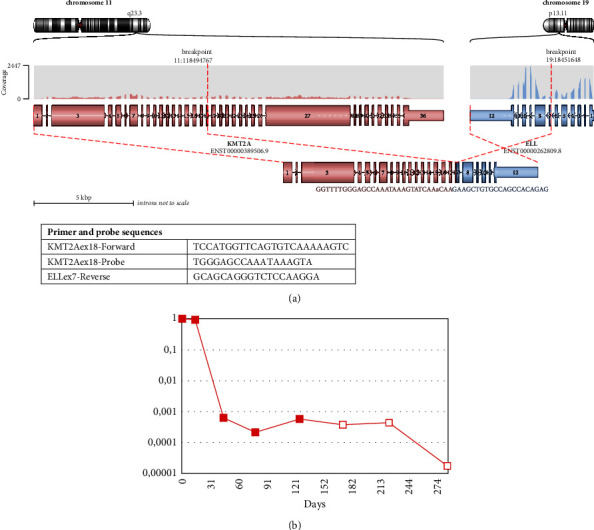
Molecular MRD monitoring via *KMT2A*::*ELL*. Transcript fusion site in the mRNA is shown below. (a) Patient specific *KMT2A*::*ELL* fusion transcript. (b) Molecular MRD monitoring over time. Red filling = bone marrow aspirate. Filled symbol = MRD detected at this level. Hollow symbol = MRD negative at this level of sensitivity.

Final cytogenetic report confirmed an abnormal clone present with a t(11;19) (q23;p13.1) translocation. Following RNA-based NGS testing, a *KMT2A*::*ELL* fusion was detected (Figure 2(a)), consistent with the t(11;19) (q23;p13.1) found in the cytogenetic study. Sequence data from this fusion were used to design a patient specific RT-qPCR assay for molecular monitoring.

Treatment according to UK paediatric Myechild AML guidelines was initiated, and the patient went in to a complete haematological remission but remained MRD positive on the basis of molecular monitoring and subsequently underwent escalation of chemotherapy to fludarabine, cytarabine, and idarubicin (FLA-IDA) and allogeneic transplant in first complete remission. As there was no leukaemia-associated immunophenotype for flow MRD monitoring, the molecular MRD monitoring using patient-specific primer/probe set for RT-qPCR was a key to track response to therapy. Follow-up bone marrow aspirates immediately before and one month after transplant were negative by molecular MRD (Figure 2(b)).

## 3. Discussion

We presented a case of a 20-month-old girl with infantile AML with a *KMT2A*::*ELL* fusion, mimicking JMML at initial presentation. Although immunophenotyping may not identify blasts in monocytic AML; in this case, the diagnosis was further blurred by the presence of a *CBL* mutation (common in JMML with a frequency of around 15% [1, 4]), which in association with some of the clinical features (age of onset, the abnormal high monocyte count, the absence of blasts, the splenomegaly and the raised HbF) raised a diagnostic dilemma. Altogether, in the absence of the identification of the *KMT2A* rearrangement in the initial work-up, the diagnostic criteria for JMML per 2016 WHO classification (being our guideline used when this patient presented) were met (Table 1) [3].

However, chromosomal rearrangements involving the *KMT2A* gene do not exist in JMML [2, 8]. Indeed, the recently updated 5th edition of the WHO Classification of Haematolymphoid Tumours includes the absence of *KMT2A* rearrangements as one of the required diagnostic criteria for JMML (Table 1, Footnote) [2, 5].

In contrast, *KMT2A* rearrangement is frequently found in AML, accounting for 15–20% of paediatric AML [6]. *KMT2A* rearrangements occur more often in children than in adults with AML and are particularly more frequent in infancy, accounting for around 50% of leukaemia cases in children under 2 years of age [6]. *KMT2A*-rearranged AML in infants can present with hepatosplenomegaly and low blast count, resembling JMML [4]. The *KMT2A::MLLT3* fusion resulting from t(9;11) (p22;q23) is found most common, representing 46% of all *KMT2A*-rearranged AML in children [6]. This type of *KMT2A*-rearranged AML is highly associated with monocytic characteristics that can mimic JMML [4]. Saad et al. described a case of AML with t(9;11) translocation, where appropriate start of treatment was delayed due to presentation masquerading as JMML [8]. Yet, over 100 different fusion partners have been identified [6] and Kanayama et al. described two different fusion partners, *KMT2A*::*SEPT6* fusion and *KMT2A*::*ELL* fusion, both cases initially resembling JMML [7].

The presence of the *CBL* mutation initially found could be seen as a red herring in our case. However, although *CBL* mutations are more commonly found in JMML, they do occur in AML and rarely in ALL [9].

It is important to point out that there was a lack of consensus regarding bone marrow morphology between reporting haematologists. In monocytic AML, promonocytes should be considered as blast equivalents. However, since the morphology and immunophenotypic characteristics of leukemic cells in acute monocytic leukaemia may overlap with cells of normal monocytic origin, the diagnosis can be challenging and morphological diagnosis requires expert operators [10]. Since the monocytic proliferation representing the acute leukaemia cells may not look like more typical myeloid blasts but just a monocytic infiltration of cells, immunophenotyping does not necessarily help.

## 4. Conclusion

In conclusion, this case highlights the importance of considering AML with *KMT2A* rearrangement as a differential diagnosis in infantile cases with atypical monocytosis, despite low or absent blast percentage. Morphological investigation, performed by an experienced haematologist, is of utmost importance since immunophenotyping is not necessarily distinguishing in cases of monocytic AML. However, morphological examination in these cases can still be difficult and cytogenetic studies with early focused FISH testing to detect *KMT2A* gene rearrangements should be immediately performed in every suspicious case to unveil a correct diagnosis. The recent updated WHO diagnostic criteria for JMML emphasize this importance of cytogenetics. Furthermore, our case also demonstrates the utility of molecular monitoring in such AML cases where a leukaemia-associated immunophenotype is not established. And in this case, this was key in identifying ongoing MRD-positive AML requiring intensification of therapy and allogeneic transplant.

## Figures and Tables

**Table 1:** Diagnostic criteria for JMML per 2016 WHO classification (guideline used when this case presented).

I: Clinical and hematologic features (all 4 features mandatory)	II: Genetic studies (1 finding sufficient)	III: For patients without genetic features, besides the clinical and hematologic features listed under I, the following criteria must be fulfilled
PB monocyte count ≥1 × 10^9^/L	Somatic mutation in *PTPN11*^∗^ or *KRAS*^∗^ or *NRAS*^∗^	Monosomy 7 or any other chromosomal abnormality or at least 2 of the following criteria:
Blast percentage in PB and BM <20%	Clinical diagnosis of neurofibromatosis type 1 or *NF1* mutation	Haemoglobin F increased for age
Splenomegaly	Germ line *CBL* mutation and loss of heterozygosity of *CBL*^†^	Myeloid or erythroid precursors on PB smear
Absence of Philadelphia chromosome (*BCR*::*ABL1* rearrangement)		GM-CSF hypersensitivity in colony assay
		Hyperphosphorylation of STAT5

^∗^Germline line mutations (indicating Noonan syndrome) need to be excluded. ^†^Occasional cases with heterozygous splice site mutations. *Note.* updates were published in the 5^th^ edition of the World Health Organization Classification of Haematolymphoid Tumors. The changes to the diagnostic criteria of JMML include (i) absence of *KMT2A* rearrangements as one of the required diagnostic criteria; (ii) elimination of monosomy 7 as a cytogenetic criterion; (iii) hypersensitivity to GM-CSF by colony assay and STAT5 hyperphosphorylation combined as one minor criterion; (iv) thrombocytopenia with hypercellular bone marrow added as one minor criterion [2].

## Data Availability

The data can be made available from the corresponding author upon reasonable request.
